# Efficient epistasis inference via higher-order covariance matrix factorization

**DOI:** 10.1101/2024.10.14.618287

**Published:** 2024-10-14

**Authors:** Kai S. Shimagaki, John P. Barton

**Affiliations:** 1 Department of Computational and Systems Biology, University of Pittsburgh School of Medicine, USA.; 2 Department of Physics and Astronomy, University of Pittsburgh, USA.

## Abstract

Epistasis can profoundly influence evolutionary dynamics. Temporal genetic data, consisting of sequences sampled repeatedly from a population over time, provides a unique resource to understand how epistasis shapes evolution. However, detecting epistatic interactions from sequence data is technically challenging. Existing methods for identifying epistasis are computationally demanding, limiting their applicability to real-world data. Here, we present a novel computational method for inferring epistasis that significantly reduces computational costs without sacrificing accuracy. We validated our approach in simulations and applied it to study HIV-1 evolution over multiple years in a data set of 16 individuals. There we observed a strong excess of negative epistatic interactions between beneficial mutations, especially mutations involved in immune escape. Our method is general and could be used to characterize epistasis in other large data sets.

## Introduction

Epistasis is common in nature and plays an important role in evolution ^1,2^. In the presence of epistasis, the fitness effects of mutations are contingent on the genetic background in which they appear, making the relationship between sequence and function complex ^3–5^. More accurate estimates of epistasis could improve our ability to predict evolution, both at the level of genetic sequences and phenotypes ^6–8^.

Enormous amounts of time-resolved sequence data have been generated in recent years, opening up the possibility of inferring epistasis from observations of evolution. Naively, we anticipate that sets of mutations that improve fitness will be found together in the same genetic sequence more often than expected by chance, while sets of deleterious mutations will be observed less frequently. However, phenomena such as genetic hitchhiking ^9^ and clonal interference ^10^ can also generate correlations between mutations that are unrelated to function. At present, a few methods exist to estimate pairwise epistatic interactions from temporal data, but computational constraints limit their applicability to small numbers of loci ^11–13^.

Here we propose an efficient method for inferring epistatic fitness that extends and vastly improves the computational efficiency of an approach developed by Sohail and collaborators ^13^. With this new approach, the required memory and computational complexity scale only quadratically with the number of loci. These improvements are due to an efficient higher-order covariance matrix factorization (HCMF) method, which allows us to analyze much larger data sets than in previous analyses.

After validating our method in simulations, we apply it to study epistasis in within-host human immunodeficiency virus (HIV)-1 evolution in a cohort of 16 individuals. Several past studies have highlighted the role of epistasis in viral evolution. Early experimental work found evidence for both synergistic ^14^ and negative ^15^ epistasis in different viruses. Epistasis has been observed in influenza and in SARS-CoV-2, especially in the context of immune evasion ^16–21^. In HIV-1, epistasis has been observed between mutations involved in drug resistance ^22–24^ and immune escape ^25,26^. Here we found a consistent pattern of negative epistasis in HIV-1, with an interaction strength that typically scales along with the fitness effects of the individual mutations. Overall, our HCMF method enables the estimation of epistasis in large data sets, and our analysis contributes to the quantification of epistasis in viral evolution.

### Epistasis inference framework

As in related work ^13,27^, our modeling framework is based on the Wright-Fisher (WF) model ^28–30^. We write the number of haploid individuals with genotype α at time t in a population as $n_{\alpha }\left(t\right)$. Given a total population size of N, we write the genotype frequency as $z_{\alpha }\left(t\right)\;=\;n_{\alpha }\left(t\right)/N$. The state of the population is described by frequencies $z\;=\;\left(z_{1},\;z_{2},\;\ldots ,\;z_{M}\right)=\;{\left(z_{\alpha }\left(t\right)\right)}_{\alpha }$ for each of the M possible genotypes. Its evolution is influenced by several factors, including mutation, recombination, and the fitness of each genotype. To model the fitness effects of individual mutations and pairwise epistasis, we assume a fitness function

(1) $$f^{\alpha }\;=\;f\left(g^{\alpha }\right)\;=\;1\;+\;\sum_{i}s_{i}g_{i}^{\alpha }\;+\;\sum_{i<j}s_{ij}g_{i}^{\alpha }g_{j}^{\alpha }.$$

In the expression above, each locus i has a corresponding selection coefficient $s_{i}$ that quantifies the fitness effect of the mutant allele at that locus and epistatic interactions $s_{ij}$ with mutant alleles at all other loci j. For simplicity, we’ve used a binary model where each allele is either wild-type (WT) or mutant, but this can easily be extended to realistic sequence models (see Methods). Following this binary assumption, there are $M\;=\;2^{L}$ possible genotypes for sequences with L loci. The $g_{i}^{\alpha }$ above are indicator functions, with a value equal to one if genotype α has a mutant allele at locus i and zero otherwise. Ultimately, our goal will be to infer the underlying fitness parameters ${\left(s_{i}\right)}_{i=1}^{L}$ and ${\left(s_{ij}\right)}_{i<j}$ from temporal genetic data.

Under the WF model, the probability of obtaining a certain distribution of genotype frequencies in the next generation $z\left(t+1\right)$, given the current distribution $z\left(t\right)$, is multinomial:

(2) $$P\left(z\left(t+1\right)|z\left(t\right);\;N,\;\theta \right)\;=\;N!\prod_{\alpha =1}^{M}\frac{p_{\alpha }{\left(z\left(t\right)\right)}^{N_{z_{\alpha }}\left(t+1\right)}}{\left[N_{z_{\alpha }}\left(t+1\right)\right]!}.$$

We use θ as a shorthand for all evolutionary parameters, including parameters describing selection and rates of mutation and recombination (Methods).

While inferring fitness parameters directly from (2) is challenging, when generation-to-generation changes in genotype frequencies are small, we can apply the simplified diffusion approximation of the WF model ^31,32^. Through the diffusion approximation, we can obtain an analytically tractable expression for the probability of an evolutionary trajectory $L\left({\left(z\left(t_{k}\right)\right)}_{k=1}^{K}\right)$, which we refer to as the path likelihood ^13,27^ (Methods). This allows us to compute the fitness parameters (including individual selection coefficients $s_{i}$ and pairwise epistatic interactions $s_{ij}$) that best fit a data set of sequences collected over time.

To express the results, it’s useful to define a new vector $s\;=\;\left(s_{1},\;s_{2},\;\ldots ,s_{L},s_{1,2},s_{1,3},\;\ldots ,s_{L-1,L}\right)\;=\;{\left(s_{e}\right)}_{e}$. This vector combines both selection coefficients for individual mutations and pairwise epistatic interactions, with a generalized index e that runs over both single loci $\left(1,2,\;\ldots \;,L\right)$ and pairs of loci $\left(\left(1,2\right),\;\left(1,3\right),\;\ldots \;,\left(L\;-\;1,L\right)\right)$. Similarly, we can define a mutant allele frequency vector $x\;=\;\left(x_{1},\ldots ,x_{L},x_{1,2},\;\ldots ,x_{L-1,L}\right)$, where

(3) $$x_{i}\;=\;\sum_{\alpha }g_{i}^{\alpha }z_{\alpha }\;x_{i,j}\;=\;\sum_{\alpha }g_{i}^{\alpha }g_{j}^{\alpha }z_{\alpha }.$$

Higher order mutant allele frequencies (e.g., $x_{i,j,k}$) are defined similarly.

The fitness parameters $\hat{s}$ that maximize the path likelihood, together with a Gaussian prior distribution (or equivalently, ridge regression penalty) for the selection coefficients and epistatic interactions, are then given by ^13,27^

(4) $$\hat{s}\;=\;{\left(C^{\mathrm{int}}\;+\;\gamma I\right)}^{-1}\;\left(\Delta x^{\mathrm{int}}\;-\;u^{\mathrm{int}}\;-\;v^{\mathrm{int}}\right).$$

In this expression, the observed net allele frequency change over the observation period is

$$\Delta x^{\mathrm{int}}\;=\;\sum_{k=0}^{K-1}\Delta x\left(t_{k}\right)\;=\;\sum_{k=0}^{K-1}\left(x\left(t_{k+1}\right)\;-x\left(t_{K}\right)\right)\;\;\;\;\;\;\;\;\;=x\left(t_{K}\right)\;-\;x\left(t_{0}\right),$$

while $u^{\mathrm{int}}$ and $v^{\mathrm{int}}$ represent expected cumulative frequency changes due to mutation and recombination, respectively. Explicit derivations and definitions for these terms are given in Methods. $C^{\mathrm{int}}$ is the allele frequency covariance matrix integrated over time, and γ quantifies the width of the prior distribution for the selection coefficients and epistatic interactions.

Although (4) is complicated, it can be interpreted intuitively. Essentially, (4) states that allele frequency change that is *not* explained by the forces of mutation or recombination is evidence of selection. The sign and magnitude of inferred selection depend on the net change in allele frequencies (how much, quantified by $\Delta x^{\mathrm{int}}$, and how fast, quantified by the diagonal part of $C^{\mathrm{int}}$) as well as the effects of genetic background (quantified by the off-diagonal terms of $C^{\mathrm{int}}$).

## Results

### Factorization of higher-order integrated covariance matrix and efficient inference framework

While (4) provides a powerful expression to simultaneously estimate the fitness effects of mutations and pairwise epistasis from temporal genetic data, it faces a serious computational limitation. The covariance matrix $C^{\mathrm{int}}$ is $D\;=\;qL\left(qL+1\right)/2-$ dimensional, where q is the number of alleles at each locus. The computational complexity of inverting the covariance matrix thus scales as $O\left({\left(qL\right)}^{6}\right)$, with memory costs related to the storage of covariance matrix entries scaling as $O\left({\left(qL\right)}^{4}\right)$. For data sets with hundreds or thousands of loci, simply storing the covariance matrix in memory becomes challenging.

We developed an efficient and generic method to resolve the major computational bottleneck hindering the application of this approach to larger data sets. The key idea of our approach is to exploit the regular structure of the covariance matrix, allowing us to factorize the matrix and perform calculations in a lower-dimensional space without any loss of information. Specifically, we can write the integrated covariance matrix in terms of a rectangular matrix with dimensions D×d , which depends on the number of unique sequences in the data set. Writing the number of unique sequences in the data set at time $t_{k}$ as $d_{k}$, we have $d\;=\;\sum_{k=0}^{K}d_{k}\;+\;K\;-\;1$. As we will show below, d may be multiple orders of magnitude smaller than D for relevant data sets of interest, allowing this factorization to dramatically speed up analyses.

In our analysis, we integrate the covariance matrix over the evolution using linear interpolation between sampling times, which mitigates periods of sparse sampling ^13,27^. The integrated covariance matrix with linear interpolation can be factorized as follows (see Methods for details):

(5) $$C^{\mathrm{int}}\;=\;\sum_{k=0}^{K-1}\Delta t_{k}\frac{C\left(t_{k}\right)+C\left(t_{k+1}\right)\;}{2}\;\;\;\;\;\;\;\;\;\;\;\;\;\;\;+\sum_{k=1}^{K-1}\Delta t_{k}\frac{\Delta x\left(t_{k}\right)\Delta x{\left(t_{k}\right)}^{⊤}\;}{6}\;=\sum_{k=0}^{K}\sum_{\alpha }\xi_{\alpha }\left(t_{k}\right)\;\xi_{\alpha }{\left(t_{k}\right)}^{⊤}\;+\;\;\sum_{k=1}^{K-1}\xi \left(t_{k}\right)\xi {\left(t_{k}\right)}^{⊤}\;=\;:\Xi \Xi^{⊤}.$$

The ξ vectors are defined as

(6) $$\xi_{\alpha }\left(t_{k}\right)\;=\;\left\{\begin{array}{ll} \sqrt{\frac{z_{\alpha }\left(t_{0}\right)\Delta t_{0}}{2}}\left(\sigma^{\alpha }\;-\;x\left(t_{0}\right)\right) & k\;=0\\ \sqrt{\frac{z_{\alpha }\left(t_{k}\right)\left(\Delta t_{k}+\Delta t_{k-1}\right)}{2}}\left(\sigma^{\alpha }\;-\;x\left(t_{k}\right)\right) & 0\;<\;k\;<K\\ \sqrt{\frac{z_{\alpha }\left(t_{K}\right)\Delta t_{K-1}}{2}}\left(\sigma^{\alpha }\;-\;x\left(t_{K}\right)\right) & k\;=\;K, \end{array}\right.\;\xi \left(t_{k}\right)\;=\;\sqrt{\frac{\Delta t_{k}}{6}}\Delta x\left(t_{k}\right),$$

where $\Delta t_{k}\;=\;t_{k+1}\;-\;t_{k}$, $z_{\alpha }\left(t_{k}\right)$ is the frequency of genotype α in the data at time $t_{k}$, and $\sigma^{\alpha }$ is a D-dimensional vector with entries

(7) $$\sigma_{e}^{\alpha }\;=\left\{\begin{array}{ll} g_{i}^{\alpha } & \mathrm{for}\;e\;\leq \;L\\ g_{i}^{\alpha }g_{j}^{\alpha } & \mathrm{for}\;e\;>\;L. \end{array}\right.\;$$

More complex covariance interpolation using spline curves ^33^ can also be expressed in a form similar to (5).

Using the factorized Ξ matrix (5), we can rewrite the equation for the estimated selection coefficients and epistatic interactions (4) as

(8) $$\hat{s}\;=\;\gamma^{-1}\left(\Delta {\tilde{x}}^{\mathrm{int}}\;-\;\Xi \Delta \eta \right),$$

with

(9) $$\Delta {\tilde{x}}^{\mathrm{int}}\;=\;\Delta x^{\mathrm{int}}\;-\;u^{\mathrm{int}}\;-\;v^{\mathrm{int}},\;\Delta \eta \;=\;{\left(\Xi^{⊤}\Xi \;+\;\gamma I\right)}^{-1}\;\left(\Xi^{⊤}\Delta {\tilde{x}}^{\mathrm{int}}\right).$$

Critically, computing (8) is far less computationally intensive than (4) when D≫d, as the matrix to be inverted in (8) is only d×d. In total, the computational complexities of calculations in (8) are: matrix-vector products of ΞΔη and $\Xi^{⊤}\Delta {\tilde{x}}^{\mathrm{int}}$ take $O\left(dD\right)$; matrix-matrix product of $\Xi^{⊤}\Xi$ requires $O\left(d^{2}D\right)$; solving the equation for Δη without directly solving its inverse is smaller than $O\left(d^{2+\omega }\right)$, with ω a small positive number 0<ω≤1, depending on linear optimization solvers.

This substantial computational reduction was achieved by implicitly computing the integrated covariance matrix without ever storing the covariance matrix itself. Therefore, our epistasis inference scheme is more efficient and scalable as the computational complexity scales only linearly with D (and thus quadratically with L). In comparison, even naive selection inference without epistasis scales as $D^{3}$. The expression for the selection coefficients in (8) uses no approximations. Thus, its solution is exact in the diffusion limit ^13^.

For simplicity, we initially assumed the same regularization γ for selection and epistasis. However, we have also generalized our approach so that the regularization values $\gamma_{e}$ can differ and implemented this in our code (Methods). While our analysis considers only pairwise epistatic interactions, one could further extend the fitness function to consider even higher-order interactions. For p-way epistatic interactions, the computational complexity would become $O\left(dD\right)$ with $D\;=\;\sum_{l=1}^{p}q^{l}\left(\begin{array}{l} L\\ l \end{array}\right)$.

### HCMF substantially reduces computational costs

To assess the efficiency of HCMF, we simulated population evolution under the WF model using different numbers of loci, ranging from L=50to1600. We used a constant population size of $N\;=\;10^{3}$, a mutation rate of $\mu \;=\;10^{-3}$, and a recombination rate of $r\;=\;10^{-4}$ per site per generation. Our simulations ranged over 2000 generations, with virtual samples collected for inference every 10 generations. We used a fitness landscape in which 25% of mutations were beneficial $\left(s_{i}\;=\;0.03\right)$, 25% were deleterious $\left(s_{i}\;=\;-0.03\right)$, and 50% were neutral $\left(s_{i}\;=\;0\right)$. Similarly, 25% of all pairs of sites were randomly selected to have positive/negative epistatic interactions ($s_{ij}\;=\;0.03\;\mathrm{or}\;-0.03$, respectively), with the remaining 50% of the possible epistatic interactions set to zero. To ensure sufficient sampling to measure typical results, we performed 500 simulations for each condition.

**Fig. 1.**
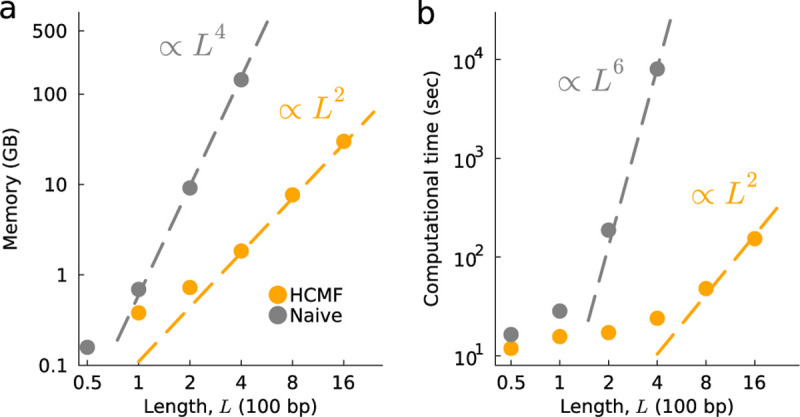
HCMF substantially reduces the required memory size and computational time. **a**, Required memory size versus number of loci L (measured in 100 bp). The required memory size of the naive method scales as $O\left(L^{4}\right)$, while our method reduces it to $O\left(L^{2}\right)$. For the naive method, we did not consider L>400 due to computational constraints. **b**, Required computational time (in seconds) versus number of loci. As anticipated, the computational time of the naive method and HCMF scale by $O\left(L^{6}\right)$ and $O\left(L^{2}\right)$, respectively.

Since the size of the covariance matrix increases quadratically with sequence length, the required memory size of the naive approach increases as $O\left(L^{4}\right)$. However, memory requirements only scale as $O\left(L^{2}\right)$ for the HCMF method (Fig. 1a). HCMF also dramatically reduces the run time of the inference, scaling as $O\left(L^{2}\right)$ compared to $O\left(L^{6}\right)$ for the naive approach (Fig. 1b). For example, for L=400, HCMF is 10^4^ times faster than the naive approach. This computational advantage should further increase for larger sequence lengths. As noted above, since the HCMF approach involves no approximations, the selection coefficients and epistatic interactions inferred by this approach match the ones from the naive method exactly within machine precision.

### Importance of higher-order covariance information for inferring epistasis

One of the main barriers to inferring epistatic interactions via (4) is computing and inverting the integrated covariance matrix. The HCMF approach we have developed offers one solution to this problem. However, one could also simplify (4) by neglecting the off-diagonal terms of the covariance matrix. This greatly reduces the computational burden of the problem, but neglects important information about linkage disequilibrium that could inform the inference of selection coefficients and epistatic interactions. We refer to this approximation as the independent model (analogous to the single locus model of ref. ^27^).

We performed additional simulations to compare the accuracy of the HCMF method, which includes higher-order covariance information, and the independent model, which does not. These simulations were performed with the same parameters as in the previous section, using L=50 loci and sparser epistatic interactions. Here we chose a random set of L/2=25 pairs of sites to have positive epistatic interactions $\left(s_{ij}\;=\;0.03\right)$, L/2 pairs with negative interactions $\left(s_{ij}\;=\;-0.03\right)$, and set the remaining epistatic interactions to zero.

**Fig. 2.**
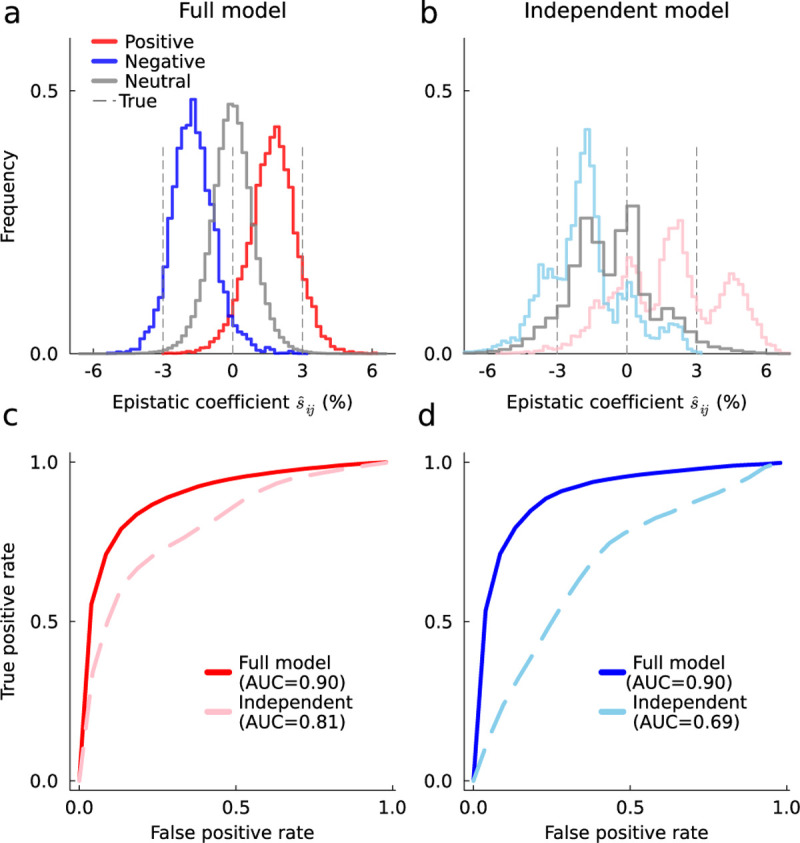
Higher-order covariance information improves the inference of epistasis. Distribution of inferred epistasis using the full model inferred via HCMF (**a**), which includes higher-order covariance information, and using the independent model (**b**), in which off-diagonal terms of the covariance matrix are set to zero. **c**, Receiver operating characteristic (ROC) curve for identifying positive epistasis. The area under the curve (AUC) value is 0.90 for the full model, while it drops to 0.81 for the independent model. **d**, Analogous ROC and AUC values for identifying negative epistasis. The AUC values are 0.90 and 0.69 for full and independent models, respectively.

The inferred epistatic interactions using the full model with HCMF are much closer to the true ones than those inferred with the independent model (Fig. 2a–b). For the full model, the distribution of inferred positive/neutral/negative epistatic interactions is roughly normal, with peaks that can easily be distinguished from one another. In contrast, the epistatic coefficients inferred using the independent model are distributed much more broadly and irregularly. While positive and negative interactions can, on average, still be distinguished from one another using the independent model, it is more difficult to do so. To quantify this difference, we computed the receiver operating characteristic (ROC) curve and area under the curve (AUC) for identifying positive (Fig. 2c) and negative (Fig. 2d) epistatic interactions using the full and independent models. Thus, by all metrics we find that the inclusion of higher-order covariance information improves the ability to identify epistatic interactions from data.

### Modeling epistasis improves the inference of selection coefficients

**Fig. 3.**
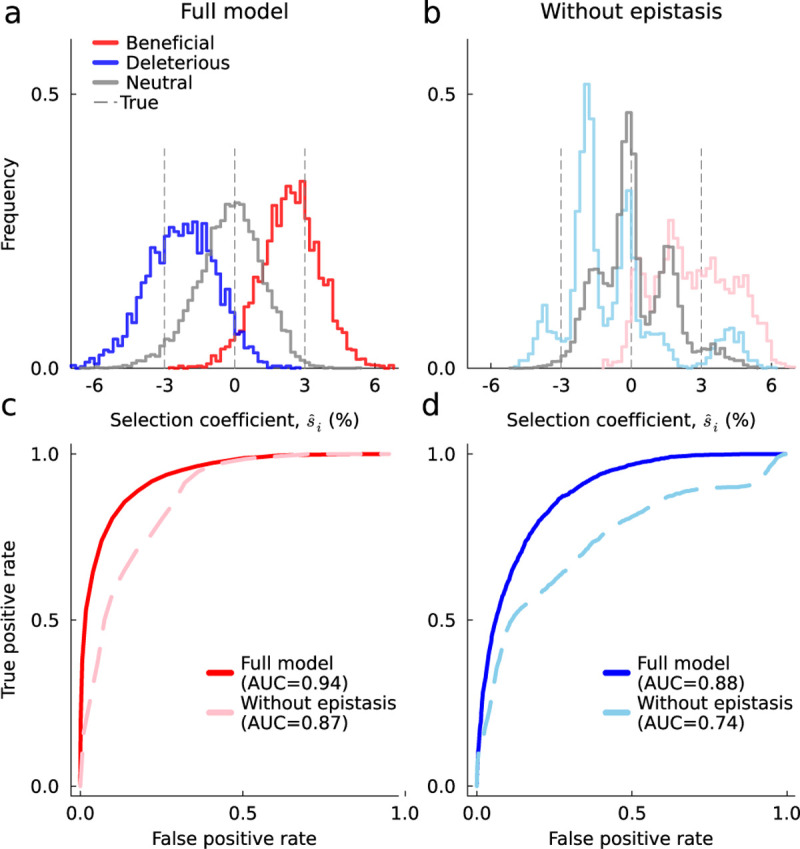
Modeling epistasis improves the inference of additive selection coefficients. Distribution of selection coefficients inferred with the full model via HCMF (**a**) and a simpler model with no epistatic interactions (**b**). When epistasis is present, including it in the model also improves estimates of selection coefficients. **c**, ROC curves and their AUC values for identifying positive selection coefficients. The AUC value of the full model is 0.94, while the AUC value of the model without epistasis drops to 0.87. **d**, Analogous ROC and AUC values for identifying deleterious selection coefficients. The AUC values are 0.88 and 0.74 for the full model and the model without epistasis, respectively. Simulation parameters are the same as in Fig. 2.

An alternative approach to reducing the computational costs of (4) is to use a simpler fitness landscape, such as a purely additive one with all $s_{ij}\;=\;0$. This assumption may be especially appropriate for analyzing highly similar sequences. However, in models with substantial, strong epistatic interactions, omitting epistasis could skew fitness estimates (Fig. 3a–b). In these conditions, inference using models with epistasis yields more accurate estimates of individual selection coefficients and improves the detection of beneficial and deleterious mutations (Fig. 3c–d).

### Joint inference from multiple replicates

Some evolution experiments, such as deep mutational scanning studies ^34,35^, have multiple independent replicates collected under the same conditions. Using our approach, we can estimate selection coefficients and epistatic interactions that best explain the data across all replicates, as shown in prior work ^13,36,37^. To demonstrate this phenomenon in a challenging setting for inference, we increased the density of epistatic interactions to 50%, with half set to be positive $\left(s_{ij}\;=\;3\%\right)$ and half negative $\left(s_{ij}\;=\;3\%\right)$. In this case, epistatic effects dominate the fitness function. With a single replicate, the AUC values were 0.82 (0.81) for identifying beneficial (deleterious) selection coefficients and 0.74 (0.72) for positive (negative) epistasis. Combining data from two replicates raised the AUC to 0.93 (0.89) for selection coefficients and 0.86 (0.85) for epistasis, with further improvements as the number of replicates increases (Supplementary Fig. 1).

### Epistasis in intrahost HIV-1 evolution

As a practical application of our approach, we studied within-host HIV-1 evolution in 16 individuals who were not treated with antiretroviral drugs during the sampling time. This data set included individuals enrolled in the CHAVI 001 and CAPRISA 002 studies in the United States, Malawi, and South Africa ^38,39^. Each individual was identified shortly after HIV-1 infection, and the viral population within each individual was sampled frequently for several months to years afterward. For most individuals, the 3′ and 5′ halves of the HIV-1 genome were sequenced separately using single genome amplification methods, preserving information about linkage disequilibrium between mutations even at long distances. For two individuals, denoted CH505 and CAP256, only the HIV-1 surface protein Env was sequenced. Most data sets consisted of around 50–100 HIV-1 sequences in total for each sequencing region, collected over 5–8 time points, with several hundred polymorphic loci (see Supplementary Table 1). However, the viral population was also sequenced more deeply in a few individuals, featuring as many as 1205 HIV-1 sequences collected at 31 time points over roughly 5 years.

Using this data, we inferred selection coefficients and epistatic interactions between HIV-1 mutations for each individual and sampling region. We used prior estimates to set the mutation ^40^ and effective recombination rates ^41^ in (8). By convention, we set the selection coefficients and epistatic interactions for the transmitted/founder (TF) sequence, the natural analog of WT, to zero (Methods). Thus, fitness effects are expressed relative to the strain of the virus that originally infected each individual. In general, the ability to transform the model parameters (i.e., selection coefficients and epistatic interactions) without affecting the dynamics of the model is referred to as a gauge freedom. Choosing a specific convention for the parameters is important for comparing fitness effects in different contexts and for improving the interpretability of the model ^37,42–44^.

Here we focused specifically on epistatic interactions between nearby sites (separated by <50 bp), with distant epistatic interactions suppressed by strong regularization. There were two reasons for our focus on short-range interactions. First, due to the high effective recombination rate in HIV-1, the size of the sequencing region, and some large time gaps between samples, the expected change in correlations between mutant alleles due to recombination may violate the mathematical assumptions of the diffusion approximation, biasing our inferences for these sites. Second, short-range epistatic interactions may be of particular biological interest in HIV-1 evolution.

The accumulation of mutations within cytotoxic T lymphocyte (CTL) epitopes – linear peptides roughly 10 amino acids in length that are recognized by cytotoxic T cells – allows mutant viruses to escape from the immune system. Past work has shown that T cells are especially important in controlling HIV-1 replication ^45^, and that the virus faces significant selective pressure to escape from CTLs ^27,39,45–47^. However, because the recognition of CTL epitopes is highly specific, even one nonsynonymous mutation within the epitope can be sufficient to confer escape ^48–50^. We anticipate that this phenomenon could lead to negative epistasis between CTL escape mutations, as the fitness benefit of multiple mutations within the epitope should be lower than expected based on the beneficial effect of each individual escape mutation.

**Fig. 4.**
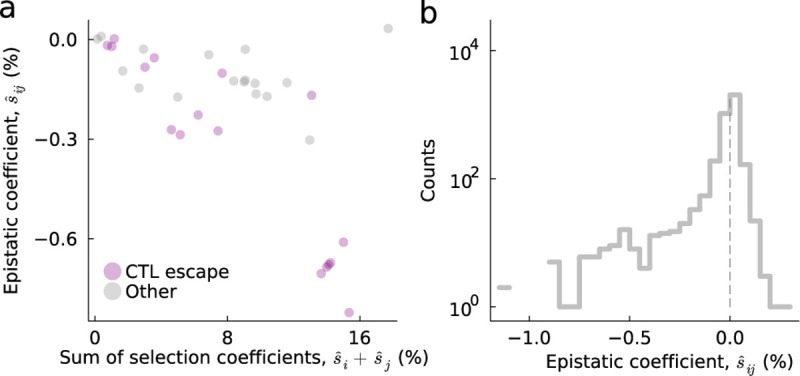
Predominance of negative epistasis between beneficial HIV-1 mutations. **a**, Comparison of inferred epistatic interactions, ${\hat{s}}_{ij}$, and the corresponding sum of individual selection coefficients, ${\hat{s}}_{i}\;+\;{\hat{s}}_{j}$, in a typical case (700010077-3; see Supplementary Fig. 2 for all individuals). Generically, we find negative correlations between inferred epistatic interactions and selection coefficients. CTL escape mutations are typically found to be both strongly beneficial and to have negative epistatic interactions with other escape mutations. **b**, Distribution of inferred epistatic interactions across all individuals. Most terms are near zero, but a few epistatic interactions are significantly negative.

While most of the epistatic interactions we inferred were very close to zero, a few were significantly negative (Fig. 4). We observed a general trend where negative epistatic interactions were more common between beneficial mutations, especially CTL escape mutations (Fig. 4). This pattern of negative epistasis between beneficial mutations, including CTL escape mutations, was robustly observed across all individuals and sequencing regions that we studied (Supplementary Fig. 2).

### Consistency with prior estimates of selection in HIV-1

Past work has studied HIV-1 evolution in part of this data set with different modeling choices, including a model with purely additive selection ^27^ and one that includes specific terms for CTL escape ^51^. Neither of these models includes pairwise epistatic interactions. Thus, we compared the selection coefficients inferred in our analysis with those from previous models to understand how the inclusion of pairwise epistasis affects the interpretation of the fitness effects of individual mutations.

**Fig. 5.**
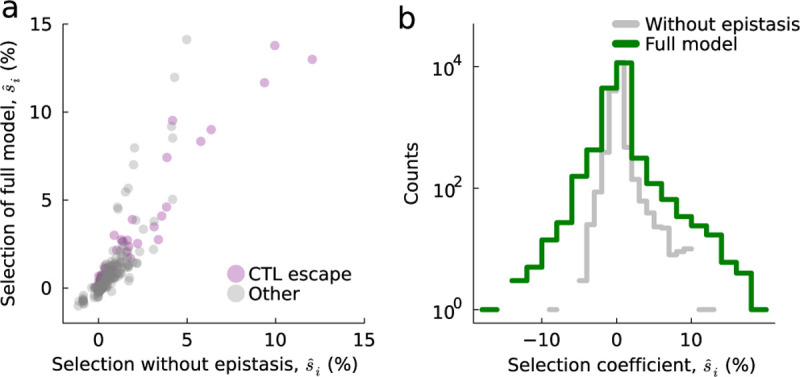
Consistency of inferred selection coefficients in models with and without epistasis. **a**, Comparison of inferred selection coefficients in models with and without epistasis in a typical case (700010077-3; see Supplementary Fig. 3 for all individuals). While the exact values differ, there is excellent general agreement between the mutations that are inferred to strongly affect fitness and those that are inferred to be nearly neutral. **b**, Distribution of selection coefficients across all individuals. Both distributions are peaked near zero, but the tails of the distributions in the full model are longer.

Figure 5a shows a typical example of the inferred selection coefficients, ${\hat{s}}_{i}$, with and without the inclusion of epistasis. Overall, we find that the inferred selection coefficients are similar to those in past models (mean Pearson’s R=0.94). In particular, all models find very strong selection for CTL escape mutations ^27,51^. As in previous work, the great majority of inferred selection coefficients are very close to zero (Fig. 5b). However, the model without epistasis also features heavier tails in the distribution of inferred selection coefficients, with more mutations inferred to have either very beneficial or very deleterious individual effects.

## Discussion

Epistasis is prevalent in nature, and has been observed to influence viral evolution ^1,15,52–54^. However, inferring epistasis from data is technically and computationally challenging. Here, we developed a new approach for the path likelihood inference framework ^13,27,37,51,55^ that greatly reduces computational costs for many data sets of interest, especially for inferring epistasis. Our key innovation was the efficient factorization of the higher-order covariance matrix, which allows us to analytically estimate selection coefficients and epistatic interactions from data without ever explicitly computing the covariance matrix or its inverse. For this reason, we referred to our method as higher-order matrix factorization (HCMF). The HCMF approach is general and can be applied under different assumptions about the structure of the fitness landscape. HCMF does not introduce any new approximations, so it suffers no loss in accuracy compared to prior approaches.

After validating our approach in simulations, we applied HCMF to study HIV-1 evolution within 16 individuals. The fitness effects of mutations that we inferred were consistent with past computational results ^27,36,47,51^ and with experimental findings. In particular, we found strong selection for mutations that allow the virus to escape from the host immune system, in agreement with a large body of experimental work and clinical observations ^25,39,45,46,56^.

In this HIV-1 data set, the distribution of epistatic interactions that we inferred was peaked near zero, but with a substantial tail of strong negative epistasis. Patterns of negative epistasis have also been observed in other viruses ^15^. Negative epistasis was especially common between CTL escape mutations, consistent with the finding that single mutations within an epitope typically already disrupt T cell recognition ^48–50^. We also observed negative epistasis between pairs of beneficial mutations more generally. This finding is consistent with more general studies that have observed decreasing effect sizes of beneficial mutations over time ^6,57–59^.

Our approach to inferring epistasis from temporal data differs from some prior methods, which used statistical models to explain correlations in protein sequences collected from many individuals or species ^22,23,26,60–64^. These models treat sequence data as samples from a static, equilibrium distribution, and interpret correlations between mutations as possible evidence for epistasis. Only a handful of methods allow for the possibility that linkage disequilibrium may arise from an underlying phylogenetic structure to the sequence data ^65,66^, or simply by chance. Nonetheless, these approaches have also been successful at tasks such as predicting the fitness effects of HIV-1 mutations in experiments ^62,63^ and the dynamics of immune escape within individual patients ^26^. In contrast to the present work, these models offer a “global” view of epistasis averaged across many related sequences.

The HCMF approach that we have developed is general. While our study focused on HIV-1, future work could be applied to other populations, including viruses like influenza and SARS-CoV-2 (ref. ^55^), experimental evolution ^37^, or bacteria ^67,68^. As one example, recent studies have suggested that epistasis plays an important role in maintaining fitness among SARS-CoV-2 Spike mutations that escape from antibodies and control receptor binding ^19,20^. More systematic studies could reveal the importance of epistasis in different aspects of SARS-CoV-2 evolution. More generally, a deeper understanding of epistasis may also improve our ability to understand and predict viral evolution.

## Supplementary Material

Supplement 1
