# Supplementary material for: Efficient epistasis inference via higher-order covariance matrix factorization
Source: bioRxiv. 2024 Oct 14:2024.10.14.618287. Preprint. [Version 1] doi: 10.1101/2024.10.14.618287 (PMC11507688; doi:10.1101/2024.10.14.618287)
Supplement: Supplement 1 [file NIHPP2024.10.14.618287v1-supplement-1.pdf]

bioRxiv preprint doi: https://doi.org/10.1101/2024.10.14.618287; this version posted October 14, 2024. The copyright holder for this preprint (which was not certified by peer review) is the author/funder, who has granted bioRxiv a license to display the preprint in perpetuity. It is made available under aCC-BY 4.0 International license.

Supplementary Information

Methods

HIV sequences

We retrieved HIV-1 sequences for individuals in this study from the Los Alamos National Laboratory (LANL) HIV Sequence Database[69] (see **Supplementary Table 1**). We processed the sequence data as described in ref.[70] to minimize the influence of sampling noise. Processing steps included 1) removing the sequences with large deletions, 2) removing sites with high gap frequencies (indicating rare insertions or potential alignment errors), and 3) eliminating time points with <4 sequences or ones that were obtained >200 days after the prior sampling time. In addition, we imputed ambiguous nucleotides using the most common nucleotides in the data at the same site within that individual.

Model

We model the evolution of a population of N individuals subject to mutation, recombination, natural selection, and genetic drift (finite population size), following the Wright-Fisher (WF) model[71–73]. We assume that all individuals are haploids. Each genotype $\boldsymbol{g} \in \mathcal{A}^L$ is a sequence of L alleles, with the alleles considered to be categorical variables $\mathcal{A} = \{$A, T, G, C, -$\}$ for DNA or $\mathcal{A} = \{$A, C, . . . , W, Y, -$\}$ for amino acids, including a gap character to represent deletions. We write the fitness of each genotype as

$$f^\alpha = f(\boldsymbol{g}^\alpha) = 1 + \sum_i s_i(g_i) + \sum_{i<j} s_{ij}(g_i, g_j)\,. \tag{S1}$$

The $s_i(a)$ are additive fitness parameters acting on each site and allele individually, while the $s_{ij}(a,b)$ are epistatic interactions between pairs of alleles a and b at sites i and j. We assume the underlying fitness parameters are constant in time, with the population's adaptation speed much faster than the rate of environmental changes. We define the probability of mutation from allele a to b as μ_{ab} per site per generation, which we assume is the same for all sites. Here we used a simple model of recombination, in which there is a probability r per site per generation for a recombination breakpoint to occur at that site. A recombinant sequence derived from two sequences $\boldsymbol{g}^\alpha$ and $\boldsymbol{g}^\beta$ with recombination breakpoint i then has the form $(g_1^\alpha, \ldots, g_i^\alpha, g_{i+1}^\beta, \ldots, g_L^\beta)$. We assume that the partner sequence β is always chosen randomly from the population with a probability proportional to the frequency of that genotype.

Let $\boldsymbol{z} \in [0,1]^M$ be the genotype frequency with the number of genotypes being M. The WF model under the fixed population size N is defined as the following multinomial process:

$$P(\boldsymbol{z}(t_k+1)|\boldsymbol{z}(t_k);N) = N! \prod_{\alpha=1}^{M} \frac{p_\alpha(\boldsymbol{z}(t_k))^{N z_\alpha(t_k+1)}}{[N z_\alpha(t_k+1)]!}\,, \tag{S2}$$

where p_α is given by

$$p_\alpha(\boldsymbol{z}) = \frac{f^\alpha z_\alpha + \sum_\beta(\mu_{\beta\alpha} z_\beta - \mu_{\alpha\beta} z_\alpha) + \sum_\gamma \sum_\beta z_\gamma [R(\alpha \mid \beta,\gamma) z_\beta - R(\beta \mid \alpha,\gamma) z_\alpha]}{\sum_\beta f^\beta z_\beta}\,. \tag{S3}$$

$R(\alpha|\beta,\gamma)$ is the probability that the recombination of genotypes β and γ results in a genotype α.

In simulations, we used $\mu_{ab} = \mu = 10^{-3}$ and $r = 10^{-4}$ per site per generation. For HIV-1 data analysis, we used mutation rates estimated from a longitudinal virus evolution study[74], along with a constant recombination rate of $r = 10^{-5}$ per site per generation, in line with past estimates of the effective recombination rate[75–79]. This choice for representing recombination in HIV-1 is a simplification. In reality, HIV-1 recombination occurs in multiple steps: first, two different viruses must coinfect the same cell. Then, genetic material from each virus can be packaged together in the same virion. When such a virion infects a new cell, recombination can occur as the viral reverse transcriptase switches between templates. Thus, the effective HIV-1 recombination rate involves both coinfection and template switching probabilities. Recent work has also shown that the effective recombination rate can increase when viral load is higher, due to increased rates of coinfection[80]. Here we applied only the simple recombination model in which probabilities of coinfection and template switching are combined into a single effective recombination rate. Future work could relax this assumption and consider the effects of time-varying recombination rates due to fluctuations in viral load.

bioRxiv preprint doi: https://doi.org/10.1101/2024.10.14.618287; this version posted October 14, 2024. The copyright holder for this preprint (which was not certified by peer review) is the author/funder, who has granted bioRxiv a license to display the preprint in perpetuity. It is made available under aCC-BY 4.0 International license.

Diffusion limit

The properties of multinomial processes lead to the following genotype average and covariance values:

$$\begin{aligned} &\sum_{\boldsymbol{z}'} z'_\alpha P(z'_\alpha \mid \boldsymbol{z}; N) = p_\alpha(\boldsymbol{z}) \\ &\sum_{\boldsymbol{z}'} (z'_\alpha - p_\alpha(\boldsymbol{z}))(z'_\beta - p_\beta(\boldsymbol{z})) P(z'_\alpha \mid \boldsymbol{z}; N) = C_{\alpha\beta}(\boldsymbol{z})/N \,, \end{aligned} \tag{S4}$$

with

$$C_{\alpha\beta} = \begin{cases} -p_\alpha p_\beta & \alpha \neq \beta \\ (1-p_\alpha)p_\alpha & \alpha = \beta \end{cases} . \tag{S5}$$

Assuming that the rates of mutation and recombination and the fitness effects of mutations are small (formally, $\mathcal{O}(1/N)$), changes in genotype frequencies are not abrupt, and we can employ the diffusion approximation[81] to simplify the WF model. This results in the following Kimura's diffusion equation (Fokker-Planck equation or Kolmogorov forward equation[82]):

$$\partial_t P(\boldsymbol{z};t) = \frac{1}{2} \sum_{\alpha,\beta} \partial_\alpha \partial_\beta C_{\alpha\beta}(\boldsymbol{z}) P(\boldsymbol{z};t) - \sum_\alpha \partial_\alpha p_\alpha(\boldsymbol{z}) P(\boldsymbol{z}) \,. \tag{S6}$$

The above equation leads to the Gaussian process with average drift and diffusion matrix (Eq. (S4)). To be more explicit, assuming $1 \ll N$ and collecting the only $\mathcal{O}(1/N)$ terms in the stochastic process, we get the following tractable expression

$$\begin{aligned} P(\boldsymbol{z}(t+\Delta t) \mid \boldsymbol{z}(t)) &\propto \exp\left(-\frac{N}{2\Delta t} \left(\boldsymbol{\Delta}\mathbf{z}(t) - \Delta t \boldsymbol{d}(\boldsymbol{z}(t)) \right)^\top C(\boldsymbol{z})^{-1} \left(\boldsymbol{\Delta}\mathbf{z}(t) - \Delta t \boldsymbol{d}(\boldsymbol{z}(t)) \right) \right) \\ d_\alpha(\boldsymbol{z}(t)) &= C_{\alpha\alpha} s_\alpha + \sum_{\beta(\neq\alpha)} C_{\alpha\beta} s_\beta + \sum_\beta (\mu_{\beta\alpha} z_\beta - \mu_{\alpha\beta} z_\alpha) + \sum_\gamma \sum_\beta z_\gamma [R(\alpha \mid \beta,\gamma) z_\beta - R(\beta \mid \alpha,\gamma) z_\alpha] \,, \end{aligned} \tag{S7}$$

Here the covariance is also taking only the $O(1/N)$ terms and scaled by N, therefore, $C_{\alpha\alpha} = z_\alpha(1-z_\alpha)$ and $C_{\alpha\beta} = -z_\alpha z_\beta$. In the main text, we gave the optimal selection coefficients $\hat{s}_i$ and epistatic interactions $\hat{s}_{ij}$ maximizing a posterior distribution over the above diffusion processes (Eq. (4)).

So far, we have discussed the diffusion process in the genotype distribution space. To make the expressions more transparent, we can project the genotype frequency dynamics onto the allele frequency space (Eq. (3)), which we describe below.

Expected frequency change due to mutation

Assuming the WF process, we can analytically estimate the expected frequency change due to mutation and recombination effects and integrate them over the generations. Define the indicator function $g^\alpha_{i,a}$ that gives one if genotype α has allele a at site i, otherwise zero, and $x_{i,a}$ is the allele frequency obtained by $\sum_\alpha g^\alpha_{i,a} z_\alpha = x_{i,a}$.

Since the mutation rate is small, effectively, no more than one mutation occurs per generation for individual sequences. Therefore, possible mutations between genotypes α and β are formally constrained by their distance such that $d_{\alpha,\beta} = L - \sum_{i,a} \delta_{g^\alpha_{i,a}, g^\beta_{i,a}} = 1$, with $\delta_{x,y}$ being Kronecker's delta. Therefore, the expected additive frequency change in allele a at site i due to mutation is:

$$\begin{aligned} u_{i,a} &= \sum_\alpha \sum_{\beta \mid d_{\alpha,\beta}=1} \sum_b g^\alpha_{i,a} g^\beta_{i,b} (\mu_{\beta\alpha} z_\beta - \mu_{\alpha\beta} z_\alpha) \\ &= \sum_{b \mid b \neq a} (\mu_{ba} x_{i,a} - \mu_{ab} x_{i,b}) \,. \end{aligned} \tag{S8}$$

Similarly, for pairwise frequencies we obtain

$$\begin{aligned} u_{ij,ab} &= \sum_\alpha \sum_{\beta \mid d_{\alpha,\beta}=1} g^\alpha_{i,a} g^\alpha_{j,b} (\mu_{\beta\alpha} z_\beta - \mu_{\alpha\beta} z_\alpha) \left(\sum_{c \mid c \neq a} g^\beta_{i,c} g^\beta_{j,b} + \sum_{c \mid c \neq b} g^\beta_{i,a} g^\beta_{j,c} \right) \\ &= \sum_c \left([\mu_{bc} x_{ij,ac} + \mu_{ac} x_{ij,cb}] - [\mu_{cb} + \mu_{ca}] x_{ij,ab} \right) . \end{aligned} \tag{S9}$$

bioRxiv preprint doi: https://doi.org/10.1101/2024.10.14.618287; this version posted October 14, 2024. The copyright holder for this preprint (which was not certified by peer review) is the author/funder, who has granted bioRxiv a license to display the preprint in perpetuity. It is made available under aCC-BY 4.0 International license.

Expected frequency change due to recombination

By symmetry, one can show that recombination has no effect on the expected change in individual allele frequencies[70]. However, correlations between mutations are naturally diluted by recombination. For pairwise frequencies, recombination decreases correlations between mutations until they become independent. One can show that the expected change in pairwise allele frequencies due to recombination is[83]

$$v_{ij,ab} = -r|i-j|(x_{ij,ab} - x_{i,a}x_{j,b})\,. \tag{S10}$$

A detailed derivation is included in ref.[83].

Maximum *a posteriori* solution over the path

Leveraging the analytically tractable transition probability under the diffusion limit (Eq. (S7)), the maximum *a posteriori* estimate of the selection coefficients and epistatic interactions is[83]

$$\begin{aligned}\hat{\boldsymbol{s}} &= \arg\max_{s} P(\boldsymbol{s}|\boldsymbol{\gamma}) \prod_{k=0}^{K} P(\boldsymbol{z}(t_{k+1}) \mid \boldsymbol{z}(t_k); \boldsymbol{s}, (\mu_{ab})_{a,b}, r) \\ &= (C^{\text{int}} + \text{diag}(\boldsymbol{\gamma}))^{-1} \left(\boldsymbol{x}(t_{K+1}) - \boldsymbol{x}(t_0) - \boldsymbol{u}^{\text{int}} - \boldsymbol{v}^{\text{int}}\right)\,.\end{aligned} \tag{S11}$$

Here $\text{diag}(\boldsymbol{\gamma})$ is a matrix with $\boldsymbol{\gamma}$ on the diagonal and zeros elsewhere. $P(\boldsymbol{s} \mid \boldsymbol{\gamma})$ represents a prior distribution for the selection and epistatic coefficients, given by the normal distribution

$$P(\boldsymbol{s} \mid \boldsymbol{\gamma}) \propto \exp\left(-\frac{1}{2}\boldsymbol{s}^\top \text{diag}(\boldsymbol{\gamma})\boldsymbol{s}\right)\,. \tag{S12}$$

The expected net frequency change due to mutation and recombination, denoted by $\boldsymbol{u}^{\text{int}}$ and $\boldsymbol{v}^{\text{int}}$, are defined in the equation Eq. (S8), Eq. (S9) and Eq. (S10), respectively. Further reduction based on the HCMF method is explained in the main text. We provide the expression of the factorized covariance matrix in the following section.

Representing the integrated covariance matrix with linear interpolation by a low-rank matrix

In this section, we show the expression of the integrated covariance matrix with piece-wise linear interpolation is given as the integration of covariance with a piece-wise constant interpolation and a sum of rank-one matrices.

By integrating them over the time t, we get the (time-) integrated covariance matrix. More specifically, we consider the following linear interpolation, such that

$$\begin{aligned} C^{\text{int}} &= \sum_{k=0}^{K-1} \Delta t_k \int_0^1 d\tau C^{[k,k+1]}(\tau) \\ C^{[k,k+1]}_{e,f}(\tau) &= x^{[k,k+1]}_{ef}(\tau) - x^{[k,k+1]}_{e}(\tau)\, x^{[k,k+1]}_{f}(\tau) \\ x^{[k,k+1]}_{e}(\tau) &= (1-\tau)x_e(t_k) + \tau x_e(t_{k+1})\,. \end{aligned} \tag{S13}$$

It is straightforward to check that the following expression is identical to the diagonal of the integrated covariance matrix with the piece-wise linear interpolation given in ref.[70]:

$$\begin{aligned} &\frac{\Delta t_0}{2} C_{ii}(t_0) + \frac{\Delta t_{K-1}}{2} C_{ii}(t_K) + \sum_{k=1}^{K-1} \frac{\Delta t_k + \Delta t_{k-1}}{2} C_{ii}(t_k) + \sum_{k=1}^{K-1} \frac{\Delta t_k}{6} (\Delta x(t_k))^2 \\ &= \sum_{k=0}^{K-1} \frac{\Delta t_k}{2} \Big(x_i(t_k)(1-x_i(t_k)) + x_i(t_{k+1})(1-x_i(t_{k+1})) \Big) + \sum_{k=1}^{K-1} \frac{\Delta t_k}{6} (x_i(t_{k+1}) - x_i(t_k))^2 \\ &= \sum_{k=0}^{K-1} \Delta t_k \left(\frac{x_i(t_k) + x_i(t_{k+1})}{2} - \frac{x_i(t_{k+1})(x_i(t_k) + x_i(t_{k+1}))}{3} - \frac{x_i(t_k)^2}{3} \right) \\ &= \sum_{k=0}^{K-1} \Delta t_k \left(\frac{(3 - 2x_i(t_k))(x_i(t_k) + x_i(t_{k+1}))}{6} - \frac{x_i(t_k)^2}{3} \right) = (C^{\text{int}})_{ii}\,. \end{aligned} \tag{S14}$$

For the off-diagonal case, the pairwise-frequency term $x_{ij}(t_k)$ is linear in time, and the result of the integral with the linear interpolation is the same as the integral with the piecewise-constant interpolation. Therefore, we explicitly write only the

bioRxiv preprint doi: https://doi.org/10.1101/2024.10.14.618287; this version posted October 14, 2024. The copyright holder for this preprint (which was not certified by peer review) is the author/funder, who has granted bioRxiv a license to display the preprint in perpetuity. It is made available under aCC-BY 4.0 International license.

integrals that are non-linear in time:

$$
\begin{aligned}
&\frac{\Delta t_0}{2} x_i(t_0)x_j(t_0) + \frac{\Delta t_{K-1}}{2} x_i(t_K)x_j(t_K) + \sum_{k=1}^{K-1} \frac{\Delta t_k + \Delta t_{k-1}}{2} x_i(t_k)x_j(t_k) - \sum_{k=1}^{K-1} \frac{\Delta t_k}{6} \Delta x_i(t_k)\Delta x_j(t_k) \\
&= \sum_{k=0}^{K-1} \Delta t_k \left(\frac{x_i(t_k)x_j(t_k) + x_i(t_{k+1})x_j(t_{k+1})}{2} - \frac{(x_i(t_{k+1}) - x_i(t_k))(x_j(t_{k+1}) - x_j(t_k))}{6} \right) \\
&= \sum_{k=0}^{K-1} \Delta t_k \left(\frac{x_i(t_k)x_j(t_k) + x_i(t_{k+1})x_j(t_{k+1})}{3} + \frac{x_i(t_k)x_j(t_{k+1}) + x_i(t_{k+1})x_j(t_k)}{6} \right) \\
&= -(C^{\text{int}})_{ij} + (\text{integrated pairwise frequency matrix})\,.
\end{aligned}
\tag{S15}
$$

By summarizing these equations, we represent the integrated covariance matrix as:

$$
C^{\text{int}} = \frac{\Delta t_0}{2} C(t_0) + \frac{\Delta t_{K-1}}{2} C(t_K) + \sum_{k=1}^{K-1} \frac{t_{k+1} - t_{k-1}}{2} C(t_k) + \sum_{k=1}^{K-1} \frac{\Delta t_k}{6} \boldsymbol{\Delta x}(t_k) \boldsymbol{\Delta x}(t_k)^\top , \tag{S16}
$$

which we can readily factorize by a matrix Ξ such that $C^{\text{int}} = \Xi\Xi^\top$. The size of the matrix Ξ is $D \times d$, where $d = \sum_{k=0}^{K} d(t_k) + K - 1$ with $d(t_k)$ denoted as a rank of $C(t_k)$. In most of the evolutionary data, the size of the higher-order covariance matrix is much larger than the effective matrix rank size; hence, typically $d \ll D$.

Inferring fitness parameters from multiple replicate trajectories

In cases where multiple ensembles of trajectories evolve under similar conditions, it is natural to extend the path likelihood to multiple ensembles. Suppose there are Q replicates, let q be the index of the qth replicate, $(t_k)_{k=1}^{K_q}$ be a set of sampling time-steps for the qth replicate, and $\boldsymbol{x}^q(t_k)$ be the set of single and pairwise frequencies for the qth replicate.

The maximum path likelihood solution using Q replicates can be expressed as[83]

$$
\begin{aligned}
\boldsymbol{s} &= \gamma^{-1}(\boldsymbol{\Delta x} - \Xi \boldsymbol{r}) \\
\boldsymbol{r} &= \left(\Xi\Xi^\top + \gamma I \right)^{-1} \Xi^\top \boldsymbol{\Delta x}\,,
\end{aligned}
\tag{S17}
$$

where

$$
\begin{aligned}
\boldsymbol{\Delta x} &= \sum_{q=1}^{Q} \boldsymbol{\Delta x}^q \\
\Xi^\top &= \left(\Xi^{1\top}, \dots, \Xi^{Q\top} \right) \in \mathbb{R}^{D\times B}\,.
\end{aligned}
\tag{S18}
$$

B is the total number of samples across replicates over the evolution, formally, $B = \sum_q^Q B^q$, where B^q is the total number of samples of the q-th replicate over its evolution. Intuitively, the likelihood of multiple independent trajectories is equal to the product of each of their likelihoods individually.

Gauge transformation

The effects of natural selection are determined by differences in fitness values, such as the difference between the fitness of the wild type and a mutant. Shifting the fitness values globally by adding a constant, $F(\boldsymbol{g}) \leftarrow F(\boldsymbol{g}) + \text{const.}$, has no effect on fitness differences. In the additive fitness model, it is easy to see that shifting the selection coefficient at any locus by an arbitrary constant K_i does not alter the relative fitness landscape: $\sum_i \sum_a (s_i(a) - K_i)\delta_{g_i,a} = \sum_i s_i(g_i) + \text{const}$. In other words, the effective fitness parameters can be reduced to $(q-1)L$, and the degrees of freedom that can be arbitrarily adjusted without changing the overall fitness picture are L parameters. More systematic arguments under general situations exist and are known as gauge theory in physics and mathematical physics. These concepts have been applied to many genetic sequence-based inference problems[84–86], with recent reviews for gauge theory in more complex cases[87,88]. In our study, mutation effects of the wild type or TF's allele serve as reference values; therefore, considering any effects involved with TF's alleles being zeros is a reasonable choice and makes the inference results more interpretable, as inferred parameters become sparser. To fix the gauge, we employed the following gauge transformation, which is commonly used in statistical inference for genetic sequences[89,90],

$$
\begin{aligned}
s_i(a) &\leftarrow s_i(a) - s_i(a^{\text{WT}}) + \sum_{j\ (>i)} \left(s_{ij}(a, b^{\text{WT}}) - s_{ij}(a^{\text{WT}}, b^{\text{WT}}) \right) + \sum_{j\ (<i)} \left(s_{ji}(b^{\text{WT}}, a) - s_{ji}(b^{\text{WT}}, a^{\text{WT}}) \right) \\
s_{ij}(a,b) &\leftarrow s_{ij}(a,b) - s_{ij}(a^{\text{WT}}, b) - s_{ij}(a, b^{\text{WT}}) + s_{ij}(a^{\text{WT}}, b^{\text{WT}})\,,
\end{aligned}
\tag{S19}
$$

bioRxiv preprint doi: https://doi.org/10.1101/2024.10.14.618287; this version posted October 14, 2024. The copyright holder for this preprint (which was not certified by peer review) is the author/funder, who has granted bioRxiv a license to display the preprint in perpetuity. It is made available under aCC-BY 4.0 International license.

where $a^{\mathrm{WT}}, b^{\mathrm{WT}}$ are WT (i.e., TF) alleles at locus i and j, respectively. This choice of gauge ensures $s_i(a^{\mathrm{WT}}) = s_{ij}(a^{\mathrm{WT}}, b) = s_{ij}(a, b^{\mathrm{WT}}) = 0$ for all a, b.

Further compression of Ξ

Although the size of the matrix $\Xi \in \mathbb{R}^{D\times d}$ is much smaller than the size of the full covariance matrix $\in \mathbb{R}^{D\times D}$ with $d \ll D$, still keeping Ξ can be the major bottleneck. An example is HIV-1 CH848 data, where >1200 sequences were collected sequencing more than half of the HIV-1 genome. When we naively compute Ξ storing float variables, that requires more than a terabyte of memory. To further reduce the memory usage, we only consider alleles with nonzero frequency changes $\sum_k |\Delta x_i(t_k)| > 0$. This modification is straightforwardly implemented in our program.

Heterogeneous regularization

Instead of applying constant regularization across all parameters, we use a generalized heterogeneous regularization approach with the HCMF method: $\gamma \to \boldsymbol{\gamma} = (\gamma_e)_{e=1}^{D}$. By denoting $\Lambda_{\boldsymbol{\gamma}}$ as a diagonal matrix with $(\Lambda_{\boldsymbol{\gamma}})_{ef} = \gamma_e \delta_{ef}$, then the optimal fitness parameter becomes $\hat{s} = (C + \Lambda_{\boldsymbol{\gamma}})^{-1} \boldsymbol{\Delta x}$. Consequently, the expression for the efficient expression becomes:

$$\begin{aligned} \hat{\boldsymbol{s}} &= \Lambda_{\boldsymbol{\gamma}}^{-1}(\boldsymbol{\Delta x} - \Xi \boldsymbol{\Delta \eta}) \\ \boldsymbol{\Delta \eta} &= \left(\Xi^\top \Lambda_{\boldsymbol{\gamma}}^{-1} \Xi + I\right)^{-1} (\Lambda_{\boldsymbol{\gamma}}^{-1} \Xi)^\top \boldsymbol{\Delta x}. \end{aligned} \quad \textbf{(S20)}$$

Theoretical error bars

The posterior distribution is given by the Bayes' rule,

$$P(\boldsymbol{s} | (\boldsymbol{x}(t_k))_{k=0}^{K}) \propto \exp\left(-\frac{1}{2}(\boldsymbol{s} - \hat{\boldsymbol{s}})^\top \Sigma^{-1} (\boldsymbol{s} - \hat{\boldsymbol{s}})\right), \quad \textbf{(S21)}$$

which is a normal distribution for the fitness parameters $\boldsymbol{s}$ with mean $\hat{\boldsymbol{s}}$ and a precision matrix $\Sigma^{-1} = (C^{\mathrm{int}} + \gamma I)$. In a Bayesian inference framework, the uncertainty in the inferred fitness parameters is characterized by $(C^{\mathrm{int}} + \gamma I)^{-1}$. More specifically, considering the diagonal of the covariance matrix as the theoretical "error bar," the standard deviation for s_e can be given by $((C^{\mathrm{int}} + \gamma I)^{-1})_{ee}$. Let $\tilde{\boldsymbol{\xi}}_e \in \mathbb{R}^d$ for $e \in \{1, \ldots, D\}$ be row vectors for Ξ, then by exploiting the structure of the integrated covariance matrix $C^{\mathrm{int}} = \Xi\Xi^\top$, one can get

$$\mathrm{Var}(s_e) = (\gamma N)^{-1} \left(1 - \tilde{\boldsymbol{\xi}}_e^\top \left(\Xi\Xi^\top + \gamma I\right)^{-1} \tilde{\boldsymbol{\xi}}_e\right). \quad \textbf{(S22)}$$

As the inverse in (Eq. (S22)) is easier to obtain, once the inverse is obtained, the variance of each s_e should be straightforwardly obtained.

Data and code

Data and code used in our analysis is available in the GitHub repository located at `https://github.com/bartonlab/paper-hcmf`. This repository also contains scripts that can be run to reproduce our figures and analysis.

Supplementary References

69. Los Alamos National Laboratory. Hiv sequence database (2023). URL `https://www.hiv.lanl.gov`. Accessed: 703010505 and 703010848 inpatient code.
70. Sohail, M. S., Louie, R. H., McKay, M. R. & Barton, J. P. Mpl resolves genetic linkage in fitness inference from complex evolutionary histories. *Nature biotechnology* **39**, 472–479 (2021).
71. Wright, S. Evolution in mendelian populations. *Genetics* **16**, 97 (1931).
72. Fisher, R. A. *The genetical theory of natural selection: a complete variorum edition* (Oxford University Press, 1999).
73. Ewens, W. J. *Mathematical population genetics: theoretical introduction*, vol. 27 (Springer, 2004).
74. Zanini, F., Puller, V., Brodin, J., Albert, J. & Neher, R. A. In vivo mutation rates and the landscape of fitness costs of hiv-1. *Virus evolution* **3**, vex003 (2017).
75. Sabino, E. C. *et al.* Identification of human immunodeficiency virus type 1 envelope genes recombinant between subtypes b and f in two epidemiologically linked individuals from brazil. *Journal of Virology* **68**, 6340–6346 (1994).
76. Gao, F. *et al.* Unselected mutations in the human immunodeficiency virus type 1 genome are mostly nonsynonymous and often deleterious. *Journal of virology* **78**, 2426–2433 (2004).
77. Neher, R. A. & Leitner, T. Recombination rate and selection strength in hiv intra-patient evolution. *PLoS computational biology* **6**, e1000660 (2010).
78. Batorsky, R. *et al.* Estimate of effective recombination rate and average selection coefficient for hiv in chronic infection. *Proceedings of the National Academy of Sciences* **108**, 5661–5666 (2011).
79. Song, H. *et al.* Tracking hiv-1 recombination to resolve its contribution to hiv-1 evolution in natural infection. nat commun 9: 1928 (2018).
80. Romero, E. V. & Feder, A. F. Elevated hiv viral load is associated with higher recombination rate in vivo. *Molecular Biology and Evolution* **41**, msad260 (2024).
81. Kimura, M. Diffusion models in population genetics. *Journal of Applied Probability* **1**, 177–232 (1964).
82. Risken, H. & Risken, H. *Fokker-planck equation* (Springer, 1996).
83. Sohail, M. S., Louie, R. H., Hong, Z., Barton, J. P. & McKay, M. R. Inferring epistasis from genetic time-series data. *Molecular Biology and Evolution* **39**, msac199 (2022).
84. Weigt, M., White, R. A., Szurmant, H., Hoch, J. A. & Hwa, T. Identification of direct residue contacts in protein–protein interaction by message passing. *Proceedings of the National Academy of Sciences* **106**, 67–72 (2009).

bioRxiv preprint doi: https://doi.org/10.1101/2024.10.14.618287; this version posted October 14, 2024. The copyright holder for this preprint (which was not certified by peer review) is the author/funder, who has granted bioRxiv a license to display the preprint in perpetuity. It is made available under aCC-BY 4.0 International license.

85. Morcos, F. *et al.* Direct-coupling analysis of residue coevolution captures native contacts across many protein families. *Proceedings of the National Academy of Sciences* **108**, E1293–E1301 (2011).
86. Rizzato, F. *et al.* Inference of compressed potts graphical models. *Physical Review E* **101**, 012309 (2020).
87. Posfai, A., McCandlish, D. M. & Kinney, J. B. Symmetry, gauge freedoms, and the interpretability of sequence-function relationships. *bioRxiv* (2024).
88. Posfai, A., Zhou, J., McCandlish, D. M. & Kinney, J. B. Gauge fixing for sequence-function relationships. *bioRxiv* (2024).
89. Cocco, S., Feinauer, C., Figliuzzi, M., Monasson, R. & Weigt, M. Inverse statistical physics of protein sequences: a key issues review. *Reports on Progress in Physics* **81**, 032601 (2018).
90. Ekeberg, M., Lövkvist, C., Lan, Y., Weigt, M. & Aurell, E. Improved contact prediction in proteins: using pseudolikelihoods to infer potts models. *Physical Review E—Statistical, Nonlinear, and Soft Matter Physics* **87**, 012707 (2013).

bioRxiv preprint doi: https://doi.org/10.1101/2024.10.14.618287; this version posted October 14, 2024. The copyright holder for this preprint (which was not certified by peer review) is the author/funder, who has granted bioRxiv a license to display the preprint in perpetuity. It is made available under aCC-BY 4.0 International license.

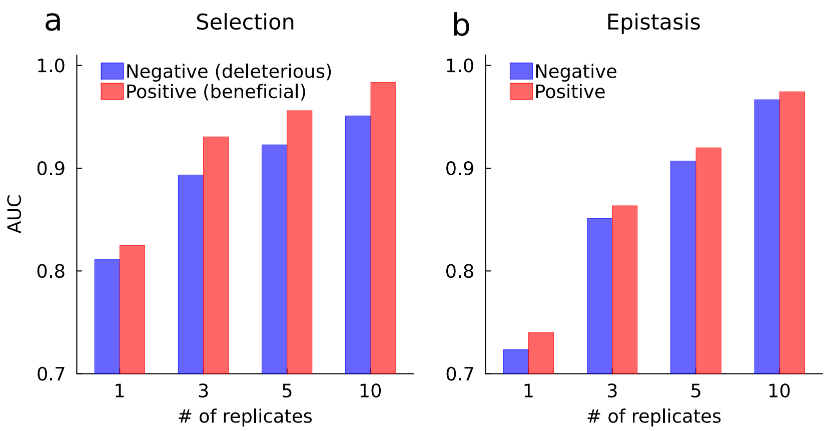

Supplementary Fig. 1. Combining evolutionary replicates improves inference accuracy. a, AUC values for identifying selection coefficients as a function of the number of replicates. When using a single trajectory, the AUC values for beneficial and deleterious coefficients are 0.82 and 0.81, respectively. The AUC values for the inferred selection coefficients increase to 0.93 and 0.89, respectively, when combining two replicates. AUC values continue to increase as the number of replicates grows, reaching 0.98 and 0.95 for beneficial and deleterious coefficients with a set of 10 replicates. **b**, AUC values for identifying epistasic interactions from a single replicate are 0.74 for positive and 0.72 for negative epistasis. Similar to the case for selection coefficients, inference accuracy steadily improves with the addition of more replicates.

bioRxiv preprint doi: https://doi.org/10.1101/2024.10.14.618287; this version posted October 14, 2024. The copyright holder for this preprint (which was not certified by peer review) is the author/funder, who has granted bioRxiv a license to display the preprint in perpetuity. It is made available under aCC-BY 4.0 International license.

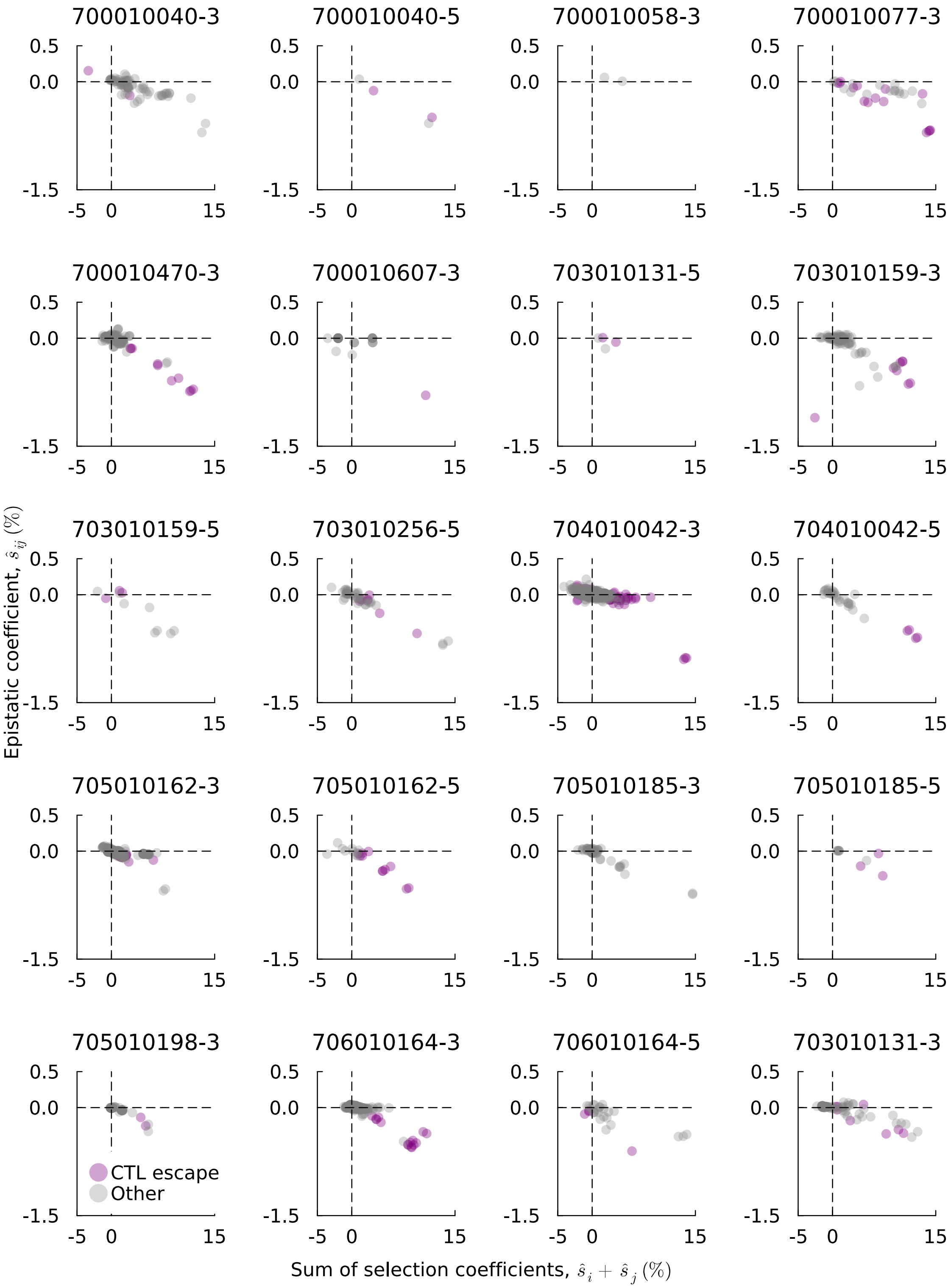

Supplementary Fig. 2. Comparison of inferred epistatic coefficients and the sum of selection coefficients. These figures are analogous to **Fig. 4a** in the main text, but for all individuals and sequencing regions that we analyzed. The tendency of strong anticorrelation between epistasis and the sum of selection coefficients is widely observed across multiple individuals. Relatively strong negative epistatic coefficients are often seen among significantly beneficial mutations, many of which are involved in CTL escape.

bioRxiv preprint doi: https://doi.org/10.1101/2024.10.14.618287; this version posted October 14, 2024. The copyright holder for this preprint (which was not certified by peer review) is the author/funder, who has granted bioRxiv a license to display the preprint in perpetuity. It is made available under aCC-BY 4.0 International license.

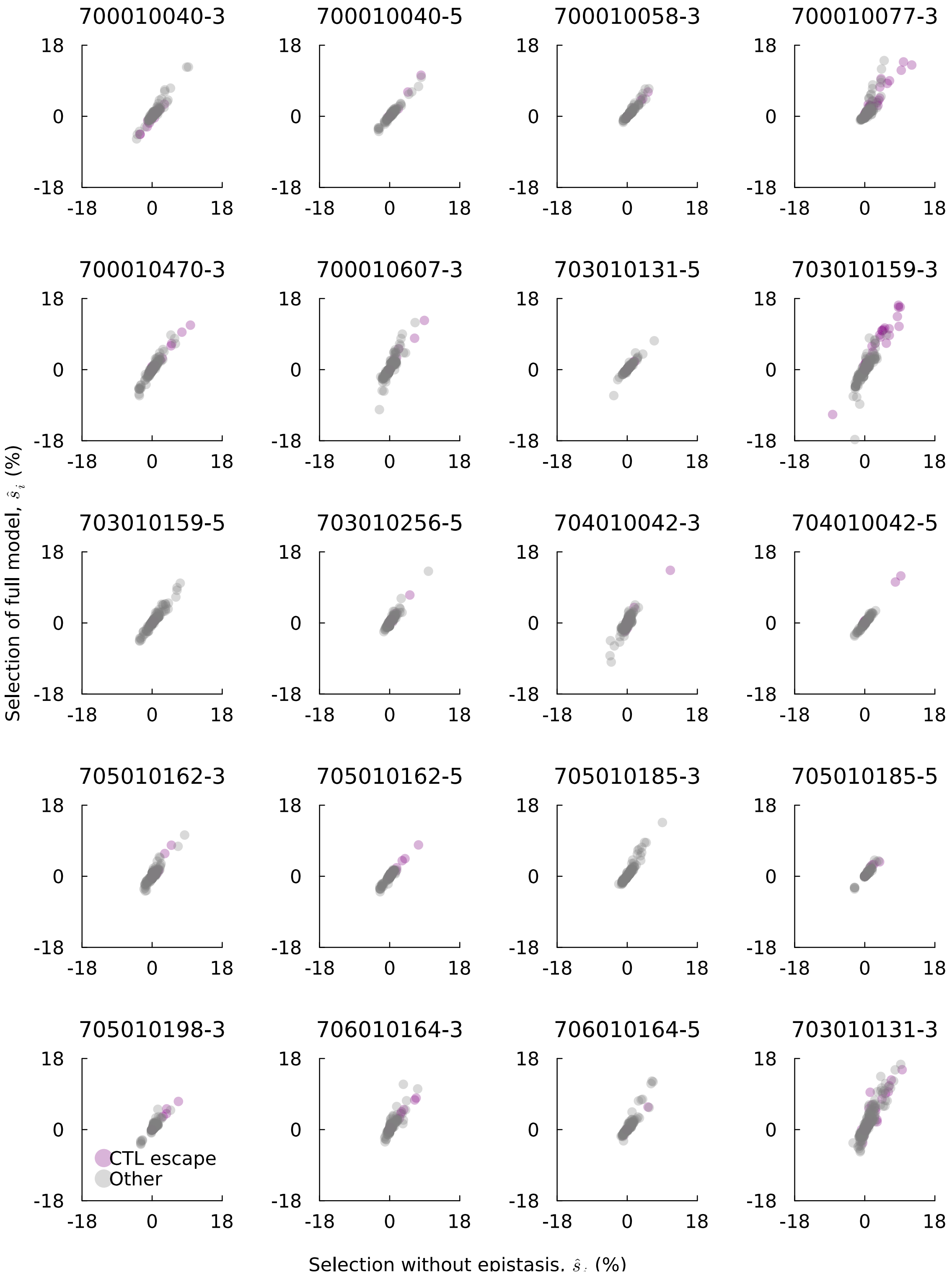

Supplementary Fig. 3. Comparison of inferred selection coefficients in models with and without epistasis. This figure is analogous to **Fig. 5a** in the main text, but across other individuals and sequencing regions that we analyzed. The inferred selection coefficients learned with epistasis are globally consistent with those learned without epistasis. Relatively strong positive selection coefficients are often involved in mutations in CTL epitopes.

bioRxiv preprint doi: https://doi.org/10.1101/2024.10.14.618287; this version posted October 14, 2024. The copyright holder for this preprint (which was not certified by peer review) is the author/funder, who has granted bioRxiv a license to display the preprint in perpetuity. It is made available under aCC-BY 4.0 International license.

ID	Length, L	Dimension, D	# of time points, K	# of sequences, N	Time (sec)	Memory (GB)
700010040-3	303	1.4×10^5	8	82	6.2	2.6
700010040-5	146	3.2×10^4	8	74	3.5	0.7
700010058-3	90	1.2×10^4	4	25	2.6	0.3
700010058-5	96	1.3×10^5	8	52	2.4	0.4
700010077-3	203	6.5×10^4	5	44	3.7	0.8
700010077-5	48	3.4×10^3	4	32	2.3	0.3
700010470-3	367	2.2×10^5	6	113	10.8	4.8
700010470-5	193	5.7×10^4	7	104	5.9	1.4
700010607-3	239	8.5×10^4	4	73	5.2	1.5
700010607-5	78	9.1×10^3	4	76	2.3	0.4
703010131-3	744	8.7×10^4	9	114	50.8	19.4
703010131-5	261	9.9×10^4	9	76	5.0	1.9
703010159-3	477	3.5×10^5	8	98	15.8	7.0
703010159-5	216	7.0×10^4	8	93	5.5	1.6
703010256-3	463	3.5×10^5	6	99	14.7	6.6
703010256-5	402	2.4×10^5	6	110	14.1	5.5
704010042-3	875	1.3×10^6	6	93	51.1	21.6
704010042-5	266	1.1×10^5	6	85	5.1	2.1
705010162-3	508	4.0×10^5	5	69	12.6	5.7
705010162-5	254	9.6×10^4	5	60	4.9	1.4
705010185-3	292	1.3×10^5	5	97	7.3	2.7
705010185-5	85	1.1×10^4	3	49	2.3	0.4
705010198-3	204	6.3×10^4	3	48	4.1	0.9
705010198-5	72	7.8×10^3	3	47	2.2	0.3
706010164-3	485	3.7×10^5	6	102	19.1	7.4
706010164-5	204	6.2×10^4	6	98	5.1	1.5
cap256-3	204	1.3×10^6	6	98	5.1	1.5
703010505	1,131	2.5×10^6	23	578	319.1	215.3
703010848	2,694	1.4×10^7	31	1,205	1,736.0	395.5

Supplementary Table 1. Computational time and required memory for inferring epistatic and selection coefficients. The table summarizes the number of polymorphic sites (length L), the effective matrix dimension to be inverted (D), the number of time points (K), as well as the required computational time and memory size for each individual. IDs consist of the patient identifier and sequencing region (5′ or 3′ end of the genome), separated by a dash. Computations were performed using a single CPU core with a single thread. For comparison, lengths of around $L \sim 200$ are already near computational limits even using a high-performance computing system.
